# c-di-GMP-related phenotypes are modulated by the interaction between a diguanylate cyclase and a polar hub protein

**DOI:** 10.1038/s41598-020-59536-9

**Published:** 2020-02-20

**Authors:** Gianlucca G. Nicastro, Gilberto H. Kaihami, André A. Pulschen, Jacobo Hernandez-Montelongo, Ana Laura Boechat, Thays de Oliveira Pereira, Caio Gomes Tavares Rosa, Eliezer Stefanello, Pio Colepicolo, Christophe Bordi, Regina L. Baldini

**Affiliations:** 1 Departamento de Bioquímica, Instituto de Química, Universidade de São Paulo, São Paulo, Brazil; 2 Instituto de Física “Gleb Wataghin”, Universidade Estadual de Campinas, Campinas, Brazil; 3 Aix Marseille Univ, CNRS, IMM, LISM, Marseille, France; 4 Present Address: Departamento de Ciencias Matemáticas y Físicas, Facultad de Ingeniería, Universidad Católica de Temuco, Temuco, Chile

**Keywords:** Bacterial adhesion, Cellular microbiology

## Abstract

c-di-GMP is a major player in the switch between biofilm and motile lifestyles. Several bacteria exhibit a large number of c-di-GMP metabolizing proteins, thus a fine-tuning of this nucleotide levels may occur. It is hypothesized that some c-di-GMP metabolizing proteins would provide the global c-di-GMP levels inside the cell whereas others would maintain a localized pool, with the resulting c-di-GMP acting at the vicinity of its production. Although attractive, this hypothesis has yet to be demonstrated in *Pseudomonas aeruginosa*. We found that the diguanylate cyclase DgcP interacts with the cytosolic region of FimV, a polar peptidoglycan-binding protein involved in type IV pilus assembly. Moreover, DgcP is located at the cell poles in wild type cells but scattered in the cytoplasm of cells lacking FimV. Overexpression of *dgcP* leads to the classical phenotypes of high c-di-GMP levels (increased biofilm and impaired motilities) in the wild-type strain, but not in a Δ*fimV* background. Therefore, our findings suggest that DgcP activity is regulated by FimV. The polar localization of DgcP might contribute to a local c-di-GMP pool that can be sensed by other proteins at the cell pole, bringing to light a specialized function for a specific diguanylate cyclase.

## Introduction

Over the past decades, (3′-5′)-cyclic diguanylic acid (c-di-GMP) has been characterized as an important second messenger in bacteria. The concentration of c-di-GMP within the cell is associated with cellular behavior: high c-di-GMP levels are linked to biofilm formation and low levels to the motile planktonic lifestyle^1,2^. This molecule is synthesized from GTP by a class of enzymes known as diguanylate cyclases (DGC) bearing a conserved GGDEF motif^3^. The c-di-GMP hydrolysis reaction is performed by phosphodiesterases (PDE) with EAL or HD-GYP domains, which cleave c-di-GMP to pGpG or GMP, respectively^4,5^. Multiple genes coding for the c-di-GMP-metabolizing proteins are found in a variety of bacterial genomes. A puzzling question in the study of c-di-GMP signaling is how the bacterial cell integrates the contributions of multiple c-di-GMP-metabolizing enzymes to mediate its cognate functional outcomes. Merritt and collaborators showed that the *P*. *aeruginosa* phenotypes controlled by two different DGC have discrete outputs despite the same level of total intracellular c-di-GMP^6^. These data support the model in which localized c-di-GMP signaling contributes to the action of proteins involved in the synthesis, degradation, and/or binding to a downstream target^2^. Studies of c-di-GMP signaling regulation during the swarmer to stalked-cell transition in *Caulobacter crescentus* also supports this hypothesis. In this dimorphic bacterium, PleD is a DGC that is inactive in swarmer cells and is activated during the swarmer-to-stalked cell differentiation^7,8^. Activation of PleD is coupled to its subcellular localization at the stalk pole, suggesting that PleD activates nearby downstream effectors involved in pole remodeling^9^. Opposite to PleD, the EAL domain protein TipF localizes at the swarmer pole, where it contributes to the proper placement of the motor organelle in the polarized predivisional cell^10^.

Even though a large body of research on c-di-GMP regulation in *P*. *aeruginosa* is available, it is still unclear whether compartmentalization of c-di-GMP signaling components is required to mediate an appropriate c-di-GMP signal transduction. The genome of *P*. *aeruginosa* strain PA14 presents forty genes coding for proteins associated with c-di-GMP metabolism^11,12^. Some of these proteins were already characterized and a few of them present a specific localization within the cell. For instance, the DGC WspR is associated with contact-dependent response to solid surfaces. Activation of the Wsp system by contact leads to the formation of subcellular clusters of WspR followed by synthesis of c-di-GMP, increasing exopolysaccharide production and biofilm formation^13^. The DGC SadC is a central player in Gac/Rsm-mediated biofilm formation^14^ and influences biofilm formation and swarming motility via modulation of exopolysaccharide production and flagellar function^15^. It was demonstrated that SadC diguanylate cyclase activity is promoted by membrane association and by direct interaction with flagellar stators disengaged from the motor^16,17^. The PDE DipA/Pch is essential for biofilm dispersion^18^ and promotes c-di-GMP heterogeneity in *P*. *aeruginosa* population^19^. This PDE is partitioned after cell division and is localized to the flagellated cell pole by the chemotaxis machinery. This asymmetric distribution during cell division results in a bimodal distribution of c-di-GMP^19^.

Previously, we demonstrated that PA14_72420 is an enzymatically active DGC that increased fitness in the presence of sub inhibitory concentrations of imipenem when overproduced in the cells^20^. Another work referred to this protein as DgcP and indicated its role in virulence^21^, but its molecular function has not yet been addressed. Thus, we decided to pursue its role by seeking for DgcP interaction partners that could participate in the same signaling pathway. DgcP was found to interact with the inner membrane protein FimV, which has a regulatory role in type IV pilus (T4P) function^22^. Moreover, we determined that DgcP localizes to cell poles in a FimV-dependent manner and is more active when the FimV protein is present. We suggest that the DgcP regulation by FimV may provide a local c-di-GMP pool at the cell pole, making this second messenger available for the c-di-GMP binding proteins that may regulate the machineries associated with the cell motility, such as the flagellum and pili.

## Results

### DgcP interacts with FimV

**Figure 1:**
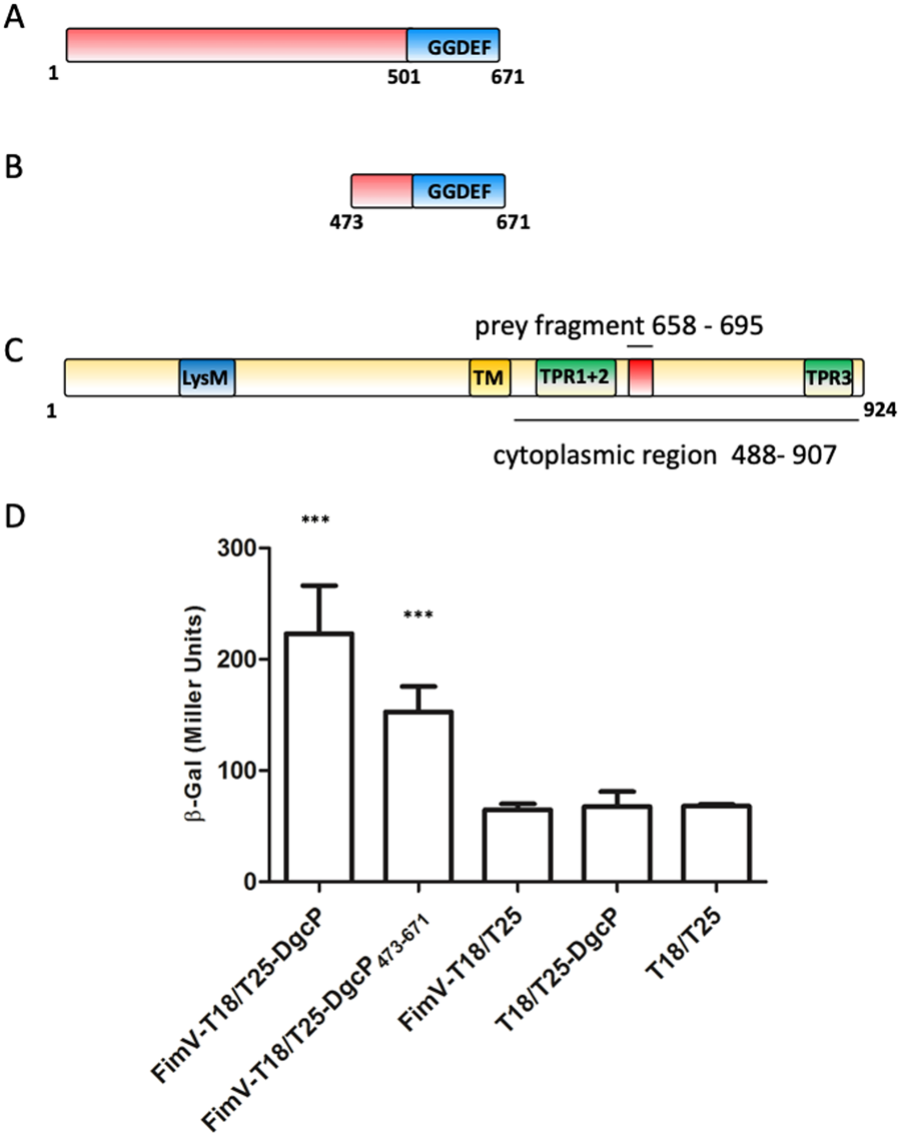
DgcP interacts with FimV. Schematic diagrams of DgcP (**A**), its C-terminus cloned in the T25-DgcP_473–671_ construct (**B**) and FimV (**C**). The GGDEF domain of DgcP is shown, as well as the transmembrane region (TM), TPR motifs and the LysM domain of FimV. The red box in FimV corresponds to the prey fragment and lines show the regions cloned to confirm the interaction. The *E*. *coli* host strain BTH101 was co-transformed with pKT25_DgcP (full length) and pUT18_FimV constructs, with the T18 tag in the C-terminal; the interactions were measured using β-galactosidase activity as a reporter (**C**). Data are the means ± SD from three replicates. ***p < 0.001.

One of the interesting paradoxes of signal transduction by c-di-GMP is the redundancy of DGCs and PDEs in bacterial cells. It has been shown that distinct phenotypes are controlled by specific DGCs or PDEs^2,6^. The specificity of DGCs and PDEs has been proposed to be related to protein localization, allowing the regulation of subcellular pools of c-di-GMP close to a target receptor^6,23^. Therefore, we sought, using the bacterial two-hybrid system (BACTH), for interaction partners of DgcP that could give a hint of DcgP function. The bait plasmid pKT25_*dgcP* was constructed with the complete *dgcP* coding region (Fig. 1A) and co-transformed in BTH101 *Escherichia coli* cells with a *P*. *aeruginosa* fragment prey library cloned in the pUT18 plasmid and derivatives^24^. About 100,000 co-transformants were obtained and 40 positive colonies identified. All positives clones were retested by individually transforming the preys cloned in pUT18 into BTH101 cells containing either the pKT25_*dgcP* or pKT25 plasmids. From the 40 clones initially obtained, 26 were confirmed and the inserts were further identified by DNA sequencing. Only two clones presented *in frame* inserts, one of them corresponding to a short peptide fragment (36 amino acids) of the cytoplasmic, C-terminal portion of FimV (Fig. 1C, red box). FimV is a large protein (924 amino acids, 97 kDa) containing a periplasmic domain with a peptidoglycan-binding LysM motif connected via a single transmembrane segment to a highly acidic cytoplasmic domain with three predicted protein-protein interaction tetratricopeptide repeat (TPR). FimV is part of the T4P secretion machinery in *Pseudomonas*^25^ and is required for localization of some T4P assembly components to the cell pole^26^. As T4P are important for initial cell attachment and the Δ*dgcP* mutant is impaired in this trait^21^, we decided to further investigate the interaction of FimV with DgcP. A construct containing the FimV cytoplasmic region was cloned into pUT18 plasmid and the GGDEF domain of DgcP was cloned in pKT25 (Fig. 1B,C). After co-transformation into BTH101 cells, we confirmed that DgcP interacts with the cytoplasmic region of FimV, and that the GGDEF domain is sufficient for such interaction (Fig. 1D)

### FimV localizes DgcP at the cell poles

**Figure 2:**
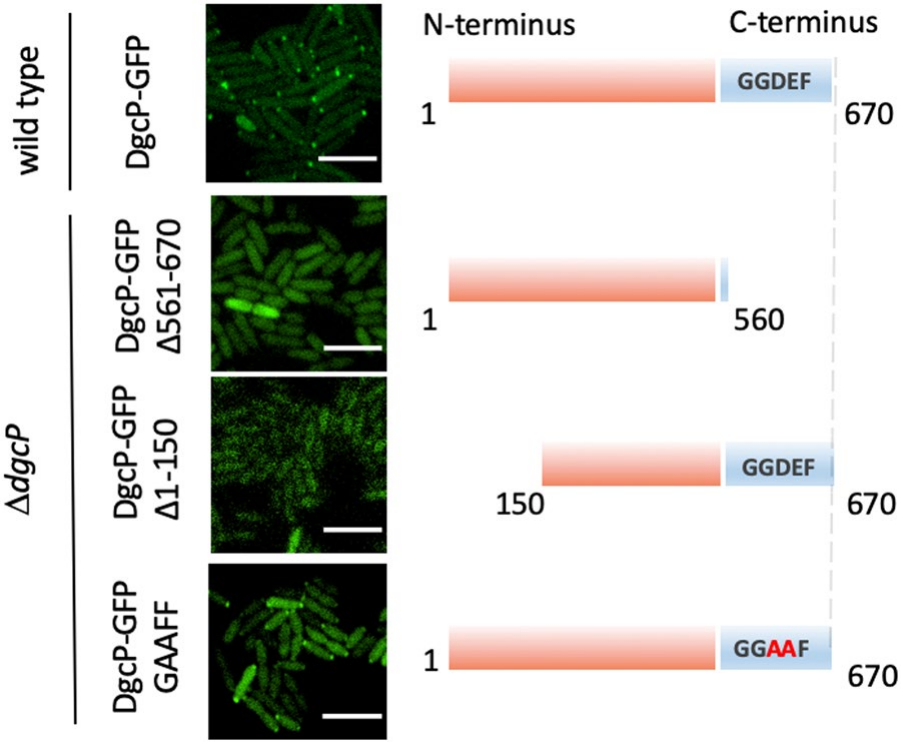
The GGDEF domain, but not its activity, is needed for DgcP localization at the cell poles. msfGFP was fusioned to wild type DgcP (1–670), to a DgcP where the conserved GGEEF motif was mutated to GGAAF and to a truncated version without the GGDEF domain (1–560) and the fusions were produced from a plasmid in PA14. Only the wild-type and GGAAF fusions localize at the cell poles. White bars represent 4 μm.

**Figure 3:**
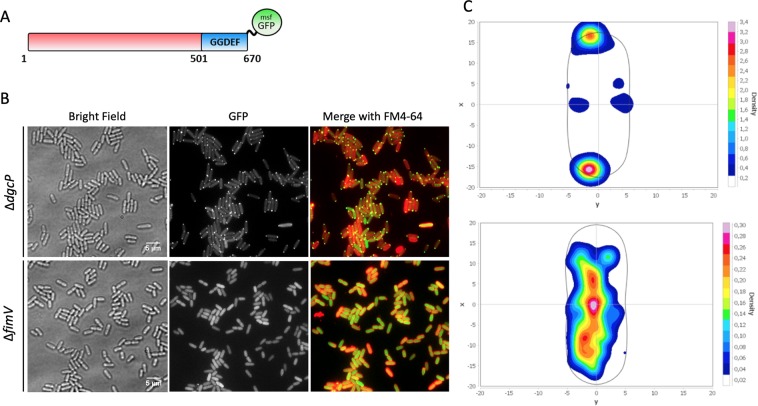
DgcP localizes at the cell poles in a FimV-dependent manner. msfGFP was fusioned to wild-type DgcP full-length and expressed in Δ*dcgP* and Δ*fimV*. (**A**) Cells were observed by bright field (left panels) and fluorescence microscopy (middle panels), and a merged image was obtained (right panels). (**B**) The intensity of the GFP fluorescence was measured in 300 cells of each strain and a heat map of DgcP_msfGFP localization was obtained with MicrobeJ, as described in the Methods section (**C**).

T4P are surface appendages that localize at the cell poles in *P*. *aeruginosa*. There are several polar localized proteins involved in the assembly and regulation of the T4P system and FimV is one of them^25–27^. Due to its interaction with FimV, we asked whether DgcP would also localize at the cell poles. Indeed, when cells overproduced DgcP fusioned to msfGFP at its C-terminus (DgcP-msfGFP), polar fluorescent foci were observed in virtually 100% of both PA14 (Fig. 2) and Δ*dgcP* strains (Fig. 3). To narrow down the DgcP region important for localization, we used different DgcP-msfGFP constructs (Fig. 2) in the Δ*dgcP* background to avoid interference of the chromosomal-encoded DgcP protein. Because there is no structural or functional information about the N-terminus region of DgcP, we ought to verify if this region could be responsible for DgcP localizazion at the poles. However, fusion proteins missing the N-terminus portion (Δ1-100, Δ1-150 and Δ1-209) are unstable (Fig. S1 and data not shown) and their localization could not be assessed. Another alternative would be that the interaction of the GGDEF domain with FimV, as uncovered in the BACTH assay, could be responsible for its localization. In fact, lack of the GGDEF domain delocalizes the fusion protein and a substitution in the catalytic motif (GGEEF to GGAAF) of DgcP has no effect. The proteins with the GGDEF domain truncated or with a point mutation present similar amounts than the wild type form (Fig. S1). These results suggest that the GGDEF domain is required for the polar localization of DgcP. In *Shewanella putrefasciens*, the GGDEF domain of the phosphodiesterase PdeB interacts with HubP, which is homologous to *P*. *aeruginosa* FimV^27^, but no structural details of this interaction are available.

The polar localization of DgcP-msfGFP is lost when this fusion was expressed in a Δ*fimV* mutant, where the fluorescence is scattered throughout the cells (Fig. 3). The pattern observed for DgcP-msfGFP is similar to that reported for FimV^26^, which is mainly in the cell poles, but also present next to the septum (Fig. 3C). This result agrees with the finding that DgcP and the cytoplasmic portion of FimV interacted in the BACTH assays. As a control, we observed that localization of the diguanilate cyclase WspR fused to msfGFP is not affected by FimV (Fig. S2).

### DgcP plays a role in biofilm formation

**Figure 4:**
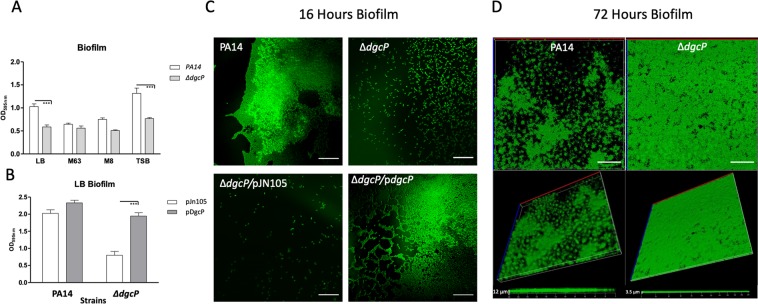
Mutation in *dgcP* affects biofilm formation. PA14 and the ∆d*gcP* strains were inoculated at OD_600_ = 0.05 in 48 well polystyrene plates with the referred media and kept at 30 °C for 16 h without agitation. The medium was discarded, and adherent cells were washed and stained with 1% crystal violet, washed and measured at OD_595_. (**A**) The same procedure was carried out for the strains overproducing DgcP in LB with 0.2% arabinose. (**B**) 3D pictures resulting from CLSM after 16 h (**C**) and 72 h (**D**) of biofilm formation in LB at 30 °C in an 8-well Lab-Tek chambered coverglass system. Data in (**A**,**B**) are the means ± SD from five replicates. ***p < 0.001. Bars in C represent 20 μm; in D, top panels, 40 μm; in lower left panel in D, 12 μm and 3.5 μm in right lower panel in D.

The role of DgcP in biofilm formation has been investigated by different groups under different conditions. Kulasekara and collaborators showed that mutation in DgcP abolished biofilm formation in LB medium^11^ and Ha and collaborators showed that a *dgcP* mutation did not affect biofilm formation in M63 minimum medium^28^. Aragon and collaborators demonstrated that deletion of *dgcP* orthologues in *Pseudomonas savastanoi* pv. savastanoi and *P*. *aeruginosa* PAK indeed decreased biofilm formation in LB^21^. Here, we confirmed that the PA14 Δ*dgcP* mutant is impaired in biofilm formation under static conditions in the rich media LB and TSB, but minor differences were observed in minimal M63 and M8 media (Fig. 4A). These results are in agreement with the differences observed by the two previous studies^11,28^. The expression *in trans* of *dgcP* restores the phenotype of biofilm defect on Δ*dgcP* (Fig. 4B,C). In LB, Δ*dgcP* was not able to form a biofilm and just a few adherent cells were observed by confocal laser scanning microscopy (CLSM) after 16 h post-inoculation, while the wild type PA14 biofilm was more developed at this time point (Fig. 4C). At 72 hours, Δ*dgcP* biofilm was thin and undifferentiated (Fig. 4D).

### DgcP has a role in twitching motility

**Figure 5:**
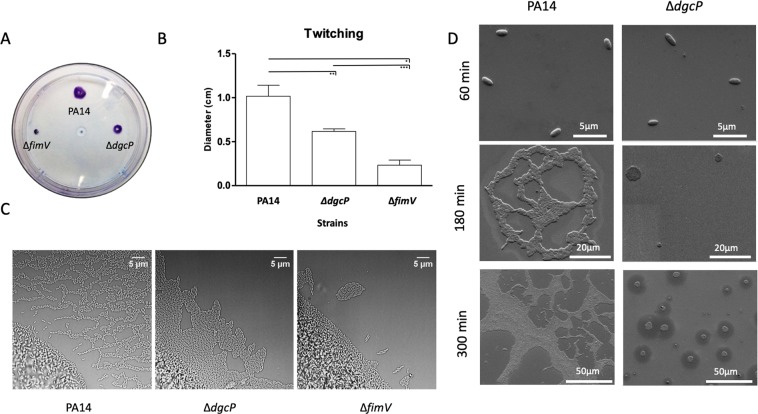
∆dg*cP* mutant presents defects related to surface behaviors. Cells were stabbed into the bottom of an agar plate by using a toothpick and incubated upright at 37 °C overnight, followed by 48 h of incubation at room temperature. The medium was discarded, and adherent cells stained with crystal violet. (**A**) Diameter of the twitching colonies was measured in triplicates. Data are the means ± SD. (**B**) Light microscopy images of PA14 and Δ*dgcP* twitching colonies. Interstitial biofilms formed at the interface between a microscope slide coated in solidified nutrient media (Gelzan Pad) after four hours of colony expansion. The Δ*fimV* mutant was used as a negative control of twitching. (**C**) A silicon slide was placed upright in a culture tube and after the different time points cells at the air-liquid interface were washed, fixed and the spread of cells during the initial stages of biofilm formation was observed by FESEM. (**D**) *p < 0.05; **p < 0.01; ***p < 0.001.

*P*. *aeruginosa* utilizes T4P to move across solid surfaces in a process known as twitching motility. As FimV is required for assembly of the T4P^25^, we decided to investigate if DgcP is important for twitching. A small portion of the outer edge of the bacterial streak was taken and stabbed into the bottom of the agar plate or placed on a thin layer of solidified media and covered with a glass coverslip. Cells were incubated and active colony expansion occurred at the interstitial interface. Twitching motility was analyzed by staining the plates with crystal violet after 16 hours (Fig. 5A,B) or by phase contrast microscopy after 4 hours of colony expansion (Fig. 5C). The Δ*dgcP* mutant presented decreased twitching motility with a less defined structure whereas PA14 presented a well-defined lattice-like network. The *fimV* mutant was not able to perform twitching motility (Fig. 5), as expected^29^. Initiation of biofilm formation was also analyzed after five hours of adhesion of cells on a silicon slide at the air-liquid interface. The cultures were adjusted to an OD_600_ = 0.05 in M63 minimum medium supplemented with glucose and casamino acids. The silicon slide was placed upright in a culture tube and cells at the air-liquid interface were analyzed by field emission scanning electron microscope (FESEM), after different time points (Fig. 5D). PA14 early biofilm presented an irregular architecture due to the motility of the initial adhering cells, but only round microcolonies that did not expand on the surface were observed for the Δ*dgcP* mutant (Fig. 5D). These results show that DgcP is important to early stages of biofilm formation and twitching motility.

### DgcP activity is FimV dependent

**Figure 6:**
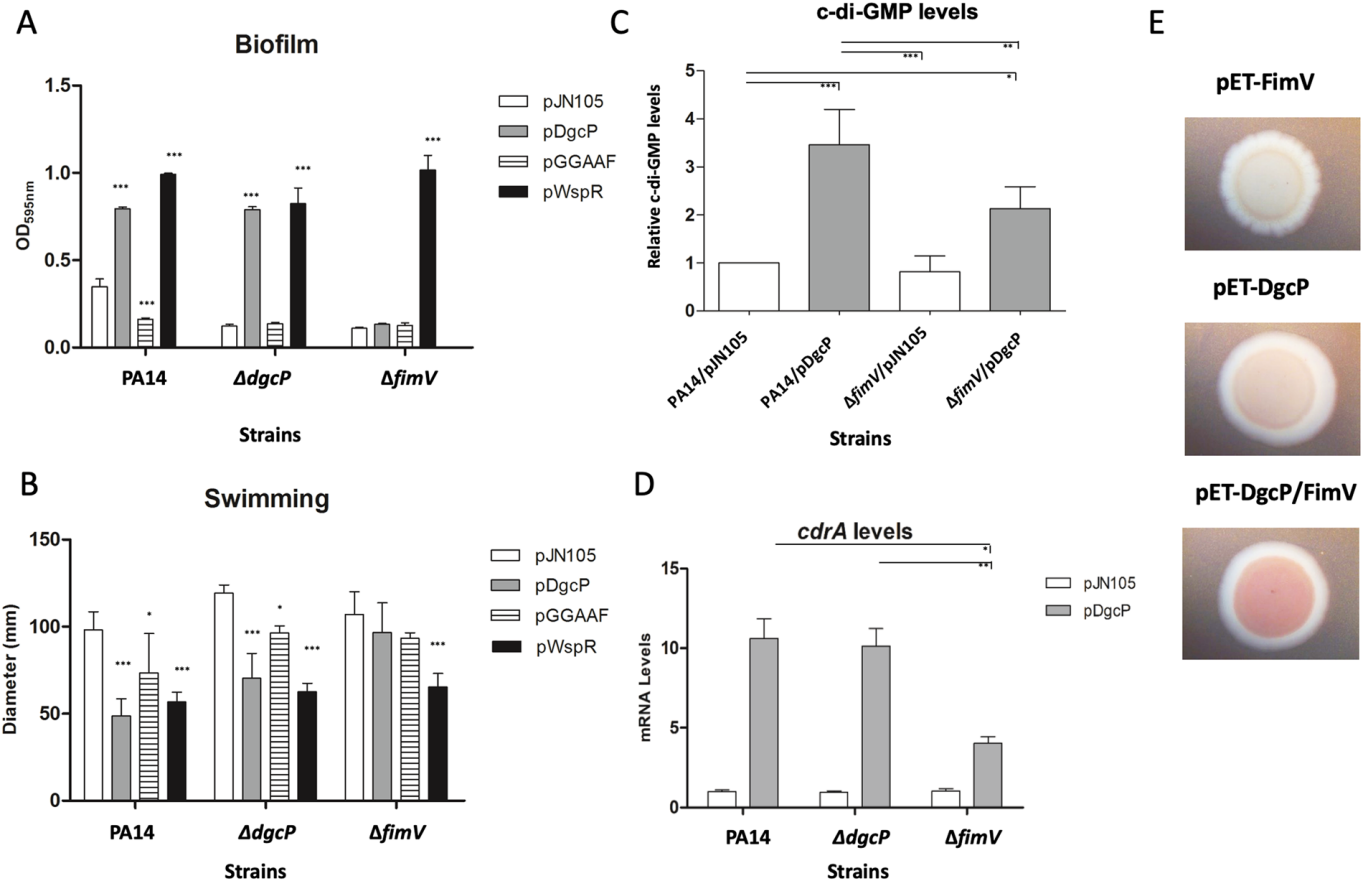
DgcP activity is FimV dependent. Full-length wild type DgcP (pDgcP) or DgcP mutated in its GGDEF domain (pDgcP-GGAAF), only DgcP C-terminal wild-type region (pDgcP-Cterm) and WspR (pWspR) were overproduced from the pJN105 vector in PA14, ΔdgcP and ΔfimV backgrounds. Biofilm (**A**), swimming motility (**B**), c-di-GMP quantitation (**C**) and cdrA mRNA relative levels (**D**) were assayed. FimV, DgcP or both were expressed in E. coli and the EPS production was assessed in Congo red plates. (**E**) Data are the means ± SD from three replicates. *p < 0.05; **p < 0.01; ***p < 0.001.

Here we observed that the FimV protein localizes the diguanylate cyclase DgcP at the cell pole (Fig. 3). Thus, we asked whether DgcP activity could be regulated by FimV. To answer this question, we overproduced the DgcP-msfGFP in the Δ*fimV* mutant, confirmed that it is stable using immunoblot against GFP (Fig. S1) and analyzed the phenotypes related to c-di-GMP. Overproducing of the DGCs DgcP and WspR fused to msfGFP in PA14 increases biofilm formation and decreases swimming motility (Fig. 6A,B), showing that these fusions are functional. Both DGCs also complement the Δ*dgcP* mutation, but DcgP-msfGFP is not able to increase biofilm formation or decrease swimming motility in the Δ*fimV* background, which is also impaired in biofilm formation (Fig. 6A). This is another indication that both proteins have complementary roles in the cells and suggests that DgcP requires FimV for full activity. This is not observed for the highly active DGC WspR-msfGFP, which has the same effect with or without FimV in the cells and therefore was used as a control. Moreover, overproduction of a mutated DgcP in the diguanylate cyclase motif (GGEEF to GGAAF) decreases biofilm formation in the wild type PA14 but has no effect on both Δ*dgcP* and Δ*fimV* backgrounds (Fig. 6). DGCs are dimeric proteins therefore the GGAAF mutation may act as a negative dominant on the wild type DgcP, either forming nonfunctional heterodimers or binding to FimV, blocking the access of the wild type DgcP in PA14.

CdrA is an extracellular protein considered as a scaffold for the biofilm extracellular matrix and transcription of *cdrA* is c-di-GMP-dependent, via FleQ.^30^ and it is widely used as a reporter of c-di-GMP levels^31^. Overproduction of DgcP-msfGFP leads to ~10-fold increased *cdrA* mRNA levels in PA14 and Δ*dgcP*, but only fourfold in the Δ*fimV* strain (Fig. 6D). Quantitation of c-di-GMP agrees with the *cdrA* expression levels (Fig. 6C). Exopolysaccharide (EPS) is also an indication of c-di-GMP levels in several bacteria^32,33^. DgcP and FimV were overproduced alone or in combination in *E*. *coli* cells and the production of EPS was assessed in Congo red plates. FimV overproduction does not result in EPS production and colonies overproducing DgcP have a pale tint. When both proteins are together in *E*. *coli*, EPS production increases, resulting in pink colonies (Fig. 6E). Altogether, our results corroborate the hypothesis that the polar localization of DgcP by FimV also regulates its activity and that DgcP may contribute to the c-di-GMP pool.

## Discussion

DgcP is a well conserved DGC protein in Pseudomonads related to plant and human infections^21^. Previously, we showed that overproduction of this protein alters biofilm formation, swimming and swarming motilities as well as fitness in the presence of imipenem, due to reduced levels of OprD^20^. However, we could not conclude that those phenotypes were specifically related to the physiological role of DgcP, because it was assumed that the overproduction of a DGC increases the global c-di-GMP levels. Therefore, we used protein-protein interactions, characterization of a deletion mutant and protein localization to look for the specific function of DgcP.

*P*. *aeruginosa* possesses polar T4P which are used for twitching motility and adhesion^34^, essential traits for mature biofilm architecture. Assembly of T4P requires FimV^25^, which shares similar domain organization with HubP, present in other proteobacteria^35,36^ and also in a number of other taxa, such as Firmicutes and Actinobacteria, as found in a homology search (not shown). These proteins, despite low overall sequence similarity, present a conserved N-terminal periplasmic domain required for polar targeting, and a highly variable C-terminal acidic cytoplasmic region, implicated in protein-protein interactions. HubP is required for polar localization of the chromosomal segregation and chemotactic machineries^35,36^. HubP of S. putrefasciens localizes the multidomain membrane protein PdeB to the flagellated pole by interacting with its GGDEF domain. PdeB, however, has a phosphodiesterase activity that is not altered by HubP, but the authors suggest that this interaction may contribute to asymmetric c-di-GMP levels in dividing cells^27^.

**Figure 7:**
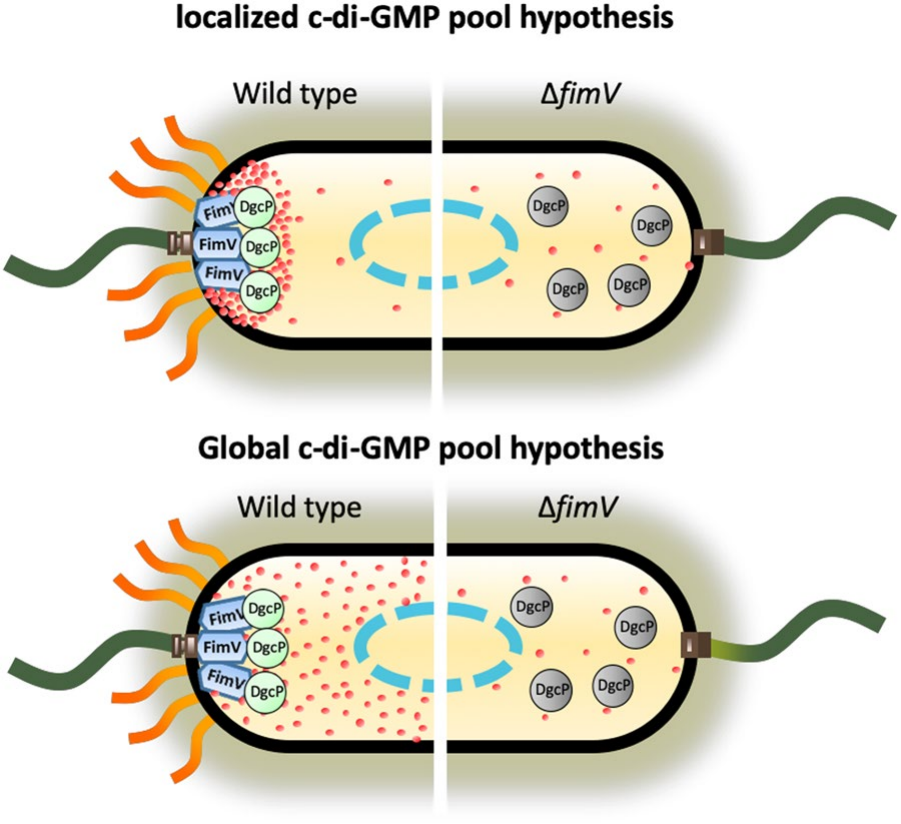
Model of FimV-dependent localization and activity of DgcP. In the PA14 wild type strain (left side of the cartoons), DgcP in its active form (green spheres) is located at the pole due to interaction with FimV (blue box) and may contribute to a local (upper panel) or global (lower panel) c-di-GMP (red dots) pool. In a ∆*fimV* background, DgcP is scattered in the cytoplasm in a less active form (gray spheres) and may have a small contribution to the c-di-GMP pool (right). Nucleoid, blue dashed line; T4P, orange wavy lines and flagella, dark green lines.

We found that DgcP is present only at the cell poles and that this pattern is dependent on FimV. We also observed that the diguanylate cyclase activity of DgcP is dependent on FimV, but the details of this activation are not understood at this point. Thus, we may envision two different scenarios. In the first, DgcP localization is important for the formation of a c-di-GMP pool at the cell poles that would assist the assembly and/or function of the T4P apparatus or other pole-localized organelles (Fig. 7, upper panel). Alternatively, the interaction of DgcP with FimV may result in a global c-di-GMP increase that could act not only at the cell poles, but in a more general fashion throughout the cell (Fig. 7, lower panel). We suggest that DgcP could be one of the sources of either localized or scattered c-di-GMP required for pilus biogenesis in *P*. *aeruginosa* and probably in other gamma-proteobacteria that carry DgcP and FimV homologs. It is worth noting that the expression of the DgcP-msfGFP constructs shown here is not subjected to endogenous regulation, since it is driven by an arabinose-inducible promoter from a multicopy plasmid. Further work is required to test whether the expression of *dgcP* from its own promoter is subjected to regulation by surface contact or other environmental cues.

Assembly of *P*. *aeruginosa* T4P requires FimX, a polarly localized c-di-GMP binding protein^37^ that has degenerate GGDEF and EAL domains and seems to be enzymatically inactive^38^. It is possible that binding of c-di-GMP to the EAL domain of FimX implicates it as an effector protein rather than a PDE, and a FimX mutant that does not bind c-di-GMP fails to activate PilB and twitching motility^39^. The FimX homolog in *Xanthomonas citri* interacts with a PilZ protein required for surface localization and assembly of pilin, but does not bind c-di-GMP^40^. *X*. *citri* PilZ subsequently interacts with PilB, an ATPase required for T4P polymerization, in a cascade of protein-protein interactions^41,42^. Remarkably, suppressor mutations in a *P*. *aeruginosa fimX* mutant that restored T4P biogenesis and partially restored twitching motility also increased c-di-GMP levels. However, the suppressor mutant cells presented peritrichous pili^37^, suggesting that, in addition to FimX, a more specific source of c-di-GMP would be needed for the correct assembly of the machinery at the cell poles. Similarly, a *P*. *aeruginosa* PilZ domain protein is also involved in the T4P-based twitching motility and does not bind to c-di-GMP, suggesting a conserved mechanism^34^. Cumulatively, these findings imply that the molecular mechanisms of pilus protrusion and retraction are regulated by local fluctuations of c-di-GMP levels. Other components of polar localized structures, such as flagella and the chemotactic machinery also bind directly or indirectly to c-di-GMP, such as FlgZ^43,44^. FlgZ is a c-di-GMP binding effector that controls *P*. *aeruginosa* motility via its interaction with the MotCD stator. Bense *et al*. described that FimV is involved in increasing the polar localized c‐di‐GMP bound FlgZ^45^, however the mechanism is still unknown and a putative contribution of DgcP is yet to be assessed.

*P*. *aeruginosa* T4P was demonstrated to be important not only to attach and move, but also to sense mechanical features of the environment. T4P sensing on solid surface increases its extension and retraction frequencies and cAMP production, leading to the upregulation of the cAMP/Vfr-dependent pathway^46^. FimV was associated with this process by interaction with FimL, a scaffold protein that connects T4P with the Chp chemosensory system via interaction with PilG and FimV^47,48^. FimV is also involved in the transcriptional upregulation of *pilY1*, and PilY1 increases the SadC DCG activity upon surface contact^49^. However, SadC presents itself in localized foci at the poles, in the middle of cells and between these two locations^16^, suggesting that it may have a broader role. Thus, we suggest that an outside signal could be sensed by T4P and transduced by FimV as described for SadC, resulting in the direct and localized activation of c-di-GMP production by DgcP, as depicted in Fig. 7. The finding that FimV-DgcP interact at the poles is an important step towards the understanding of how c-di-GMP localized pools are formed, controlling the spatial activity of target proteins.

## Methods

### Bacterial strains, plasmids and growth conditions

The bacterial strains and plasmids used in the study are described in the Supplementary Table S1. For routine cell cultures, bacteria were grown aerobically in LB broth or LB agar at 37 or 30 °C. Ampicillin (100 µg/ml), kanamycin (50 µg/ml) or gentamicin (10 µg/ml) were added to maintain the plasmids in *E*. *coli*. Carbenicillin (300 µg/ml), kanamycin (250 µg/ml) or gentamicin (50 µg/ml) were added to maintain the plasmids in *P*. *aeruginosa*. For the pJN105 related constructs, arabinose was added to cultures at 0.2% final concentration. Both M8^50^ and M63^51^ minimal salt media were supplemented with 1 mM MgSO_4_, 0.2% glucose and 0.5% casamino acids (CAA). To visualize bacterial two-hybrid interactions on solid medium, MacConkey indicator medium (Difco) supplemented with 1% maltose and 100 µM IPTG (Isopropyl β-D-1-thiogalactopyranoside), herein designated MacConkey medium, was used.

### General molecular techniques

DNA fragments were obtained by PCR using Q5 DNA polymerase (NEB). Oligonucleotide primers were purchased from Life Technologies and the sequences are listed in Table S1. PCR products of the expected sizes were purified from gels using GeneJET TM Gel Extraction Kit (Thermo Scientific), cloned using the SLIC method^52^ and transformed into *E*. *coli* DH5α. Plasmid purification was performed with GeneJET Plasmid Miniprep kit (Thermo Scientific). Sequencing was carried out using the Big Dye terminator cycle sequencing kit (Applied Biosystems) using the facility of the Departamento de Bioquímica, IQ-USP (SP, Brazil).

To construct unmarked in-frame deletions, the upstream and downstream regions of the target gene were amplified and cloned into the pEX18Ap (*fimV*) or pKNG (*dgcP*). The resulting constructs were used to delete target genes on wild type PA14 genome by homologous recombination. To construct the pDgcP plasmid the *dgcP* coding region was cloned in frame with a synthetic *msfGFP* gene into the pJN105 plasmid. The *msfGFP* codes for a N-terminal 40 amino acids spacer and a C-terminal monomeric super fold GFP. All vectors and constructs are described in more detail in Table S1.

### Biofilm assays

Three different biofilm assays were performed. The microtiter dish biofilm formation assay was performed as described^53^. The biofilms observed by confocal laser scanning microscopy (CLSM) were grown in 8-well chamber slides and stained with DAPI as described^54^ and imaged using a Zeiss - LSM 510-Meta. For the early stages of biofilm formation on silicon wafers, cultures were adjusted to an OD_600_ = 0.05 in M63 medium and transferred to a 24-well plate where silicon slides (2 × 1 cm^2^) were placed upright in each well. Before using the silicon substrates, they were previously cleaned by ultra-sonication for a period of 15 min each in acetone, isopropanol and distilled water, respectively. Slides were dried under N_2_ flow and subsequently treated with O_2_ plasma at 100 mTorr for 15 min (720 V DC, 25 mA DC, 18 W; Harrick Plasma Cleaner, PDC-32G). After 60, 180 or 300 minutes the slides were rinsed three times with water, fixed with 4% paraformaldehyde for 1 h and analyzed by field-emission scanning electron microscopy (FESEM; model F50, FEI Inspect) operated at 2 keV. Prior to examination, samples were coated with sputtered gold to prevent electrical charging.

### High-throughput two-hybrid assays

PAO1 two-hybrid library^24^ was tested against the pKT25_DgcP bait. Briefly, 25–50 ng of pUT18 library was transformed into BTH101 cells carrying the pKT25_DgcP vector and plated on MacConkey medium for 48–96 h at 30 °C. Red colonies were picked up and streaked on MacConkey plates. The positive colonies were cultivated in liquid medium, and plasmids were isolated and further analyzed. The candidate preys were retested individually for interaction with the bait by retransforming pUT18 derivative prey and pKT25 bait plasmids into BTH101 cells and also the pUT18 derivative preys and the pKT25 empty vector. The interaction was evaluated by the color of spotted co-transformants on MacConkey plates and β-galactosidase assays. Cells were grown on MacConkey plates for 96 hours and they were scrapped and suspended in 1 mL of PBS. 100 µL were used in the classical β-galactosidase assay^55^.

### Twitching assay

Macroscopic twitching assay was performed as described^28^ with minor modifications. Briefly, a colony was picked with a toothpick and stabbed at the bottom of a plate containing M8 medium supplemented with 1 mM MgSO_4_, 0.2% glucose, 0.5% casamino acids and 2.0% agar. The plates were incubated upright at 37 °C overnight, followed by 48 h of incubation at room temperature (∼25 °C). Next, the agar was removed, and the bacteria were stained with 0.1% crystal violet. Microscopic twitching assay was performed as described^56^.

### Swimming assay

Swimming assays were performed by inoculating 5 μl of a stationary phase-grown liquid cultures in M8 with 0.3% agar that were incubated for 16 h at 30 °C in a plastic bag to maintain the humidity constant^57^.

### Congo red assay

5 μl of stationary phase-grown *E*. *coli* Origami 2 (Table S1) cultures were inoculated at 1% agar plates of tryptone broth (10 g/L) containing Congo red (40 µg/mL) and Coomassie brilliant blue (20 µg/mL). The plates were incubated for 16 h at 30 °C and then for 96 hours at room temperature.

### qRT-PCR

For qRT-PCRs, total RNA was extracted with Trizol (Invitrogen), treated with DNase I (Thermo Scientific, Waltham, MA, USA) and used for cDNA synthesis with Revert Aid Premium Inverse Transcriptase (Thermo Scientific) and hexamer random primers (Thermo Scientific). cDNA was then amplified with specific primers using Maxima SYBRGreen/ROX qPCR Master Mix (Thermo Scientific) and the Step One Real Time PCR System (Applied Biosystems). *nadB* was used as internal control for normalization of total RNA levels^58^. The relative efficiency of each primer pair was tested and compared with that of *nadB* and the threshold cycle data analysis (2^−ΔΔCt^) was used^59^. All reactions were performed in triplicates, the assays were repeated at least twice using independent cultures and the results of one representative experiment are shown, with average values of technical triplicates and error bars representing standard deviation of ΔΔCt.

### Fluorescence and light microscopy

To verify the localization of DgcP_msfGFP fusions, fluorescence microscopy was performed using a Nikon Eclipse TiE microscope equipped with a 25-mm SmartShutter and an Andor EMCCD i-Xon camera. For fluorescence microscopy and bright field microscopy, a Plan APO VC Nikon 100X objective (NA = 1.4) and a Plan Fluor Nikon 40X objective (NA = 1.3) were used. For membrane staining, cells were treated with 50 µg/mL FM4-64 (Invitrogen). For phase contrast microscopy, a Plan APO λ OFN25 Nikon 100X objective (NA = 1.45) was used. All microscopy assays were performed with immobilized cells on 25% LB pads with 1.5% agarose. Image analyses were performed using the ImageJ^60^ and MicrobeJ^61^ softwares.

### c-di-GMP extraction and quantification

c-di-GMP was extracted as described by^62^ with minor modifications. 50 mL of cultures were grown in M8 medium at 37 °C and 200 rpm until reach OD_600_ = 1. Cells were collected by centrifugation resuspended in 500 µL of M8 medium with 0.6 M perchloric acid. The tubes were incubated on ice for 30 minutes and then centrifuged at 20000 *g* for 10 minutes. The pellets were used for protein quantitation and the supernatants were neutralized with 1/5 volume 2.5 M KHCO_3_. The nucleotide extracts were centrifuged again and the supernatants were stored at −80 °C. High-performance liquid chromatography (LC) was performed using the 1200 Infinity LC System (Agilent) that consists of a degasser, a quaternary pump, a thermostated autosampler (4 °C) and a temperature (30 °C)-controlled column compartment. This system was coupled to a 3200 Qtrap LC-MS/MS system equipped with an Electrospray Ionization source (ESI) (AB Sciex, USA). Analyst 1.4.2 software (AB Sciex, USA) was used to operate the equipment and calculate c-di-GMP concentrations.

Samples (injection of 10 μL) were separated by a Phenomenex Synergi Hydro-RP column (150 × 2 mm, 4 μm) using 0.1% formic acid in 15 mM ammonium acetate as mobile phase A and MeOH as mobile phase B at a flow rate of 0.3 mL min−1. The gradient program was 0 min 2% B, 0.5 min 2% B, 4.5 min 30% B, 6.0 min 80% B, 7.0 min 80% B, 7.01 min 2% B and 14 min 2% B. For quantification of c-di-GMP, the tandem mass spectrometry method multiple reaction monitoring (MRM) was used in negative mode. The following parameters were set: nebulizer, heated auxiliary and curtain gases (nitrogen) at 20, 30, 10, respectively; Turbo IonSpray voltage and temperature at −3,800 V and 250 °C, respectively; MRM transition (in m/z) 689.1 → 344.2 with a dwell time of 200 ms per transition; collision energy (CE) at −45 eV; and declustering potential at −53 V. An external standard curve was prepared for c-di-GMP in the MRM mode. The stock solution was diluted and the c-di-GMP peak area plotted against the nominal concentrations (16 to 2,000 ng mL−1).

## Supplementary information

Supp. Figures and Table.

## Data Availability

All data generated and analyzed during this study are included in this published article and its Supplementary information.
