# Supplementary material for: c-di-GMP-related phenotypes are modulated by the interaction between a diguanylate cyclase and a polar hub protein
Source: Sci Rep. 2020 Feb 20;10:3077. doi: 10.1038/s41598-020-59536-9 (PMC7033161; doi:10.1038/s41598-020-59536-9)
Supplement: Supplementary file 1 — Supp. Figures and Table. [file 41598_2020_59536_MOESM1_ESM.pdf]

c-di-GMP-related phenotypes are modulated by the interaction between a diguanylate cyclase and a polar hub protein

Gianlucca G. Nicastro, Gilberto H. Kaihami, André A. Pulschen, Jacobo Hernandez-Montelongo, Ana Laura Boechat, Thays de O. Pereira, Caio Gomes Tavares Rosa, Eliezer Stefanello, Pio Colepicolo, Christophe Bordi and Regina L. Baldini.

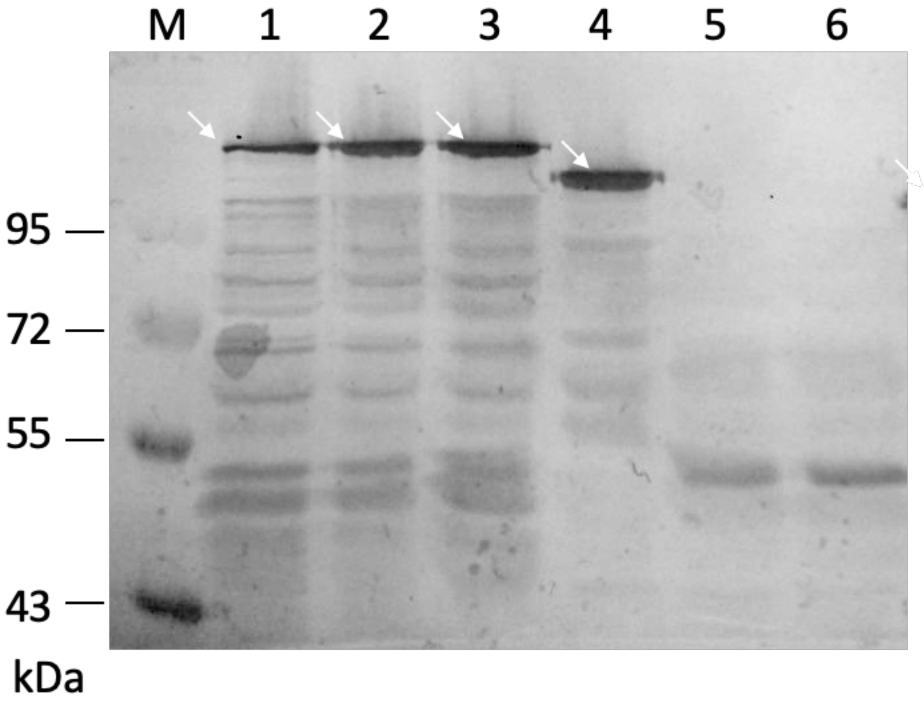

Supplementary Figure S1. DgcP-msfGFP protein fusions are detected in *P. aeruginosa* when the N-terminus is present*.* The proteins were expressed in the Δ*dgcP* (lanes 1, 3-5) or Δ*fimV* (lane 2) backgrounds in LB containing gentamycin and arabinose. Cells were lysed and similar amounts of total protein were separated by SDS-PAGE, transferred to a nitrocellulose membrane and probed with an anti-GFP antibody. White arrows show the positive GFP bands presenting the predicted molecular weights.

M, molecular weight marker

1. Δ*dgcP* /DgcP-msfGFP
2. Δ*fimV*/DgcP-msfGFP
3. Δ*dgcP* /DgcP-msfGFP GGEEF→ GGAAF
4. Δ*dgcP* /DgcP-msfGFP Δ561-670
5. Δ*dgcP* /DgcP-msfGFP Δ1-100
6. Δ*dgcP* /DgcP-msfGFP Δ1-150

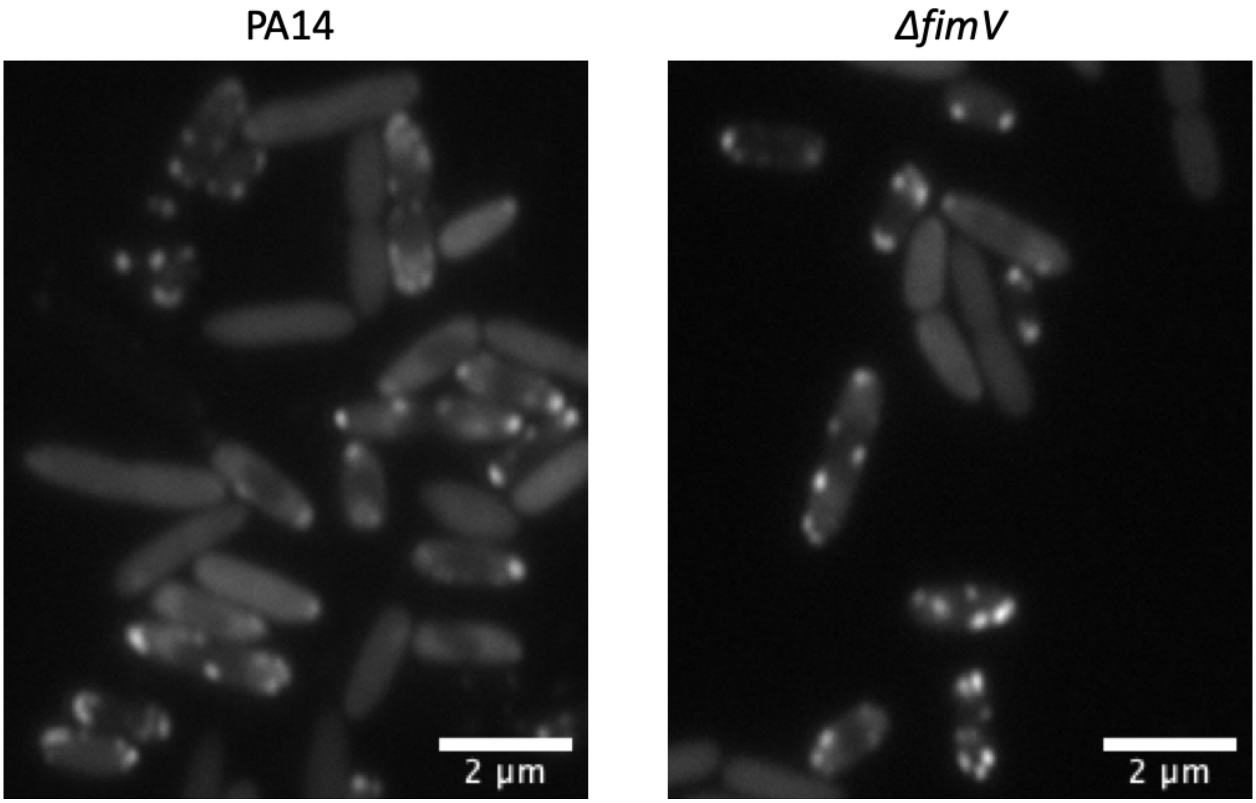

Supplementary Figure S2. FimV does not affect localization of the diguanylate cyclase WspR. msfGFP was fusioned to wild-type WspR full-length and expressed in PA14 (wild type, left panel) and Δ*fimV* (right panel). Cells were observed by bright field fluorescence microscopy as described in Material and Methods. Speckles are seen next to the cell limits, suggesting that WspR clusters localize close to the membrane in both strains.

Table S1 -Stranins plasmids and primers used in this study

Strains		
**Strains/Plasmids**	**Description**	**Reference**
***Pseudomonas aeruginosa*Strains**		
PA14	Wild-type P. aeruginosa UCBPP-PA14	(Rahme et al., 1995)
Δ*dgcP*	PA14 strain with an *in frame* deletion of *PA14_72420* gene	This Work
Δ*fimV*	PA14 strain with an *in frame* deletion of *PA14_23830* gene	This Work
***Escherichia coli* Strains**		
DH5α	*supE44 lacU169 (* 80 *lacZ* M15*) hsdR17 recA1 endA11 gyrA96 thi-1 relA1*	Invitrogen
S17-1	*prothirecA hsdR* (r - m+) Tpr Smr Kms [∧ RP4-2-Tc::Um-Km::Tn7]	(Simon et al., 1983)
BTH101	F- , *cya-99, araD139, galE15, galK16, rpsL1 (Str r), hsdR2, mcrA1, mcrB1*	(Karimova et al., 1998)
**Plasmids**		
**Plasmid**	**Description**	**Reference**
pJN105	araC-pBAD cloned in pBBR1MCS-5; GmR	(Newman and Fuqua, 1999)
pKT25	Vector encoding T25 fragment of *B. pertussis cyaA* ; Amp^R	(Karimova et al., 1998)
pUT18	Vector encoding T18 fragment of *B. pertussis cyaA* ; Amp^R	(Karimova et al., 1998)
PetDuet-1	Inducible expression vector Amp^R	Novagen
pDgcP	pJN15 plasmid containing *dgcP-msfGFP* fusion	This Work
pDgcP-Nter	pJN15 plasmid containing N term *dgcP-msfGFP* fusion	This Work
pDgcP-Cterm	pJN15 plasmid containing C term *dgcP-msfGFP* fusion	This Work
pDgcP-GGAAF	pJN15 plasmid containing *dgcP-msfGFP* fusion with GG**AA**F point mutation	This Work
pWspR	pJN15 plasmid containing *wspR-msfGFP* fusion	This Work
pET-DgcP	pEtDuet-1 plasmid containing *dgcP-* StrepTag fusion	This Work
pET-FimV	pEtDuet-1 plasmid containing *fimV-Flag* Tag fusion	This Work
pET-FimV/DgcP	pEtDuet-1 plasmid containing *fimV-Flag* Tag and dgcP-StrepTag fusions	This Work
pKT25_DgcP$_{473-671}$	pKT25 plasmid containing *cyaAT25–dgcP* (aminoacid residues 473-671) fusion	This Work
pKT25_DgcP	pKT25 plasmid containing *cyaAT25–dgcP* fusion	This Work
pUT18_FimV_long	pUT18C plasmid containing *cyaAT18–fimV Long fragment* fusion	This Work
pUT18_FimV_short	pUT18C plasmid containing *cyaAT18–fimV short* fragment fusion	This Work
**PAO1 BATCH libraries**		
pUT18CPAO1Lib	pUT18C containing PAO1 genomic library as a C-terminal T18 fusion	(Houot et al., 2012)
pUT18PAO1Lib	pUT18 containing PAO1 genomic library as an N-terminal T18 fusion	(Houot et al., 2012)
pUT18+1PAO1Lib	pUT18+1 containing PAO1 genomic library as an N-terminal T18 fusion	(Houot et al., 2012)
pUT18+2PAO1Lib	pUT18+2 containing PAO1 genomic library as an N-terminal T18 fusion	(Houot et al., 2012)

**Cloning Primers**				
Primers		Amplicon Size	Anneals to	Vector linearized by
ΔdgcP				
PA14_7242_1-2_fwd	cgtacagtagacggagtatactagt**GCTACCGCCACTACCTGA**	774 bp	PA14 gDNA	SpeI
PA14_7242_1-2_rev	gaacggacagtatga**TTCTCCAGGTACTTGTCTTTCC**			
PA14_7242_3-4_fwd	acctggagaatcata**CTGTCCGTTCCATTTCAAGG**	760 bp	PA14 gDNA	
PA14_7242_3-4_rev	tccacggactatagactatactagt **GAGGAAACGCTGGAGGAAC**			
ΔfimV				
fimV_1-2_fwd	cctgcaggtcgactctagag **TAGCTTCGATTTTTCCAGTG**	1000 bp	PA14 gDNA	PCR
fimV_1-2_rev	catttccgga **AGTGTAATCCCTTGGTTTTAATATC**			
fimV_3-4_fwd	ggattacact **TCCGGAAATGAAGCGACCCT**	1000 bp	PA14 gDNA	
fimV_3-4_rev	attcgagctcggtacccggg **GATACCGCAGATCTTGATGCG**			
PEXap _rev	**CTCTAGAGTCGACCTGCAGGC**	5838 bp	pEXap	
PEXap_fwd	**CCCGGGTACCGAGCTCGAAT**			
**pDgcP**				
pJN105_rev	**CGCTAGCCCAAAAAAACG**	6032 bp	pJN105	PCR
pJN105_fwd	**ACTAGTTCTAGAGCGGCC**			
72420_fwd	cgtttttttgggctagcg **GCGGGGATGCCGGGAGTC**	2034 bp	PA14 gDNA	
72420_rev	ccctcaagt **GCCACTTCCAGGCGGTCC**			
msfGFP_fwd	ggaagtggc**ACTTGAGGGTAGCGGACA**	838 bp	*msfGFP* synthetic gene*	
msfGFP_rev	ggccgctctagaactagt **TTATTTGTAAAGTTCATCCATTCCATG**			
**pDgcP-Cterm**				
pJN105_CTer72420_fwd	gatgccgggagtccgccatg **AGTCGCGACGACGTCCAG**	595 bp	PA14 gDNA	PCR
pJN105_CTer72420_Rev	ttgtccgctaccctcaagtg **GGATCGGTGTATTGCTTGCC**			
pjn105_7242_GFP_6896_rev	**CATGGCGGACTCCCGGCA**	6896 bp	pJN105_72420_msfGFP	
pjn105_7242_GFP_6896_fwd	**CACTTGAGGGTAGCGGACAAGG**			
**pDgcP-Nterm**				
pJN105_NTer72420_fwd	gatgccgggagtccgccatg **GAGCACGACGAGGCGCCA**	1378 bp	PA14 gDNA	PCR
pJN105_NTer72420_Rev	ttgtccgctaccctcaagtg **CCACTTCCAGGCGGTCCC**			
pjn105_7242_GFP_6870_rev	**CATGGCGGACTCCCGGCA**	6896 bp	pJN105_72420_msfGFP	
pjn105_7242_GFP_6870_fwd	**CACTTGAGGGTAGCGGACAAGG**			
**pDgcP-GGAAF**				
pJN105_rev	**CGCTAGCCCAAAAAAACG**	6032 bp	pJN105	PCR
pJN105_fwd	**ACTAGTTCTAGAGCGGCC**			
72420_fwd	cgtttttttgggctagcg **GCGGGGATGCCGGGAGTC**	1795	pJN105_72420_msfGFP	
GGAAF_rev	gacgacgaacgccgc**GCCGCCGAAACGGGCGAT**			
GGAAF_fwd	tttcggcggcgcggcg **TTCGTCGTCCTGCTCCCG**	1071	pJN105_72420_msfGFP	
msfGFP_rev	ggccgctctagaactagt **TTATTTGTAAAGTTCATCCATTCCATG**			
**pWspR**				
pjn105_GFP_rev	**CGCTAGCCCAAAAAAACG**	6870	pJN105_72420_msfGFP	PCR
pjn105_GFP_fwd	**ACTTGAGGGTAGCGGACA**			
WspR_GFP_fwd	cccgtttttttgggctagcg **CCGGAGAGAAACATGCAC**	1034	PA14 gDNA	
WspRGFP_rev	cttgtccgctaccctcaagt **ACCGGCTGTTCCATCAGG**			
**pKT25_dgcP**				
pKT25_PA14_72420_fwd	GGATCCCCAGCGCTGGAAAGACAAGTA	2361 bp	PA14 gDNA	BamHI and KpnI
pKT25_PA14_72420_rev	GGTACCGATCACGAACAGGATGATGC			

**pKT25_dgcP$_{473-671}$**				
pKT25 DgcP 473_fwd	**cagggtcgactctagaggatGCCGAGCACGAACAGGAGGT**	4400	pKT25_dgcP	PCR
pKT25 DgcP 473_rev	**ATCCTCTAGAGTCGACCCTGC**			
**pUT18_FimV_long**				
pUT18_FimV_long _fwd	ctgcaggtcgactctagaggatcct **AATGCGCAGAAAGAGAAGG**	1266 bp	PA14 gDNA	BamHI
pUT18_FimV_long _rev	ttcgagctcggtacccgggt **GTTACCTTCGGCCAGGAC**			
**pUT18_FimV_short**				
pUT18_FimV_short _fwd	ctgcaggtcgactctagaggatcctt **ATAGCTTCAGCCTCGACG**	425 bp	PA14 gDNA	BamHI
pUT18_FimV_short _rev	ttcgagctcggtacccgggt **GAGGGTGAATTCGTTGTTG**			
**pET-FimV**				
FimV_flag_fwd	actttaagaaggagatatac**ATGAATGCGCAGAAAGAGAAG**	1296	PA14 gDNA	NcoI
FimV_flag_rev	tgatggtgatggctgctgcc **TCATTTGTCGTCGTCGTCTTTATAG**			
**pET-DgcP**				
72420_Strep_fwd	gtataagaaggagatataca**ATGGCAAGCTGGAGCCACCC**	2037	PA14 gDNA	NdeI
72420_Strep_rev	tatccaattgagatctgcca **TCAGGCCACTTCCAGGCGGT**			
Real time quantitative PCR primers				
PA14_61200 up (*cdrA*)	ACAGCCAGTTCAACGACCTC	150 bp	PA14 gDNA	
PA14_61200 low (*cdrA*)	TGAAATACTCGCTGCCATTG			
nadB up	CTACCTGGACATCAGCCACA	94 bp	PA14 gDNA	
nadB low	GGTAATGTCGATGCCGAAGT			

**msfGFP* synthetic gene was kindly provided by Alexandre Bisson

Bases that annels to the template in bold and in uppercase

Bases in lowercase letters anneals to the plasmid for cloning purposes

References

Rahme LG, **Stevens EJ**, **Wolfort SF**, **Shao J**, **Tompkins RG**, **Ausubel FM**. 1995. Common virulence factors for bacterial pathogenicity in plants and animals. Science (80-) **268**:1899–1902.

Simon R, **Priefer U**, **Puhler A**. 1983. A broad host range mobilization system for in vivo genetic engineering: transposon mutagenesis in gram negative bacteria. Biotechnol (N Y) **1**:784–790.

Karimova G, **Pidoux J**, **Ullmann A**, **Ladant D**. 1998. A bacterial two-hybrid system based on a reconstituted signal transduction pathway. Proc Natl Acad Sci U S A **95**:5752–5756.

Newman JR, **Fuqua C**. 1999. Broad-host-range expression vectors that carry the L-arabinose-inducible Escherichia coliaraBAD promoter and the araC regulator. Gene1999/02/19. **227**:197–203.

Houot L, **Fanni A**, **de Bentzmann S**, **Bordi C**. 2012. A bacterial two-hybrid genome fragment library for deciphering regulatory networks of the opportunistic pathogen Pseudomonas aeruginosa. Microbiology2012/05/26. **158**:1964–1971.
